# HER2-specific chimeric antigen receptor-T cells for targeted therapy of metastatic colorectal cancer

**DOI:** 10.1038/s41419-021-04100-0

**Published:** 2021-11-27

**Authors:** Jie Xu, Qingtao Meng, Hao Sun, Xinwei Zhang, Jun Yun, Bin Li, Shenshen Wu, Xiaobo Li, Hongbao Yang, Haitao Zhu, Michael Aschner, Michela Relucenti, Giuseppe Familiari, Rui Chen

**Affiliations:** 1 School of Public Health, Kunming Medical University, Kunming, 650500 China; 2 Key Laboratory of Environmental Medicine Engineering, Ministry of Education, School of Public Health, Southeast University, Nanjing, 210009 China; 3 School of Public Health, Advanced Innovation Center for Human Brain Protection, Capital Medical University, Beijing, 100069 China; 4 Beijing Key Laboratory of Environmental Toxicology, Capital Medical University, Beijing, 100069 China; 5 Nanjing Municipal Center for Disease Control and Prevention, Nanjing, 210003 China; 6 Center for Drug Safety Evaluation and Research, China Pharmaceutical University, Nanjing, 211198 China; 7 Colorectal Cancer Center, Department of General Surgery, Jiangsu Cancer Hospital, Cancer Research Institute, Cancer hospital of Nanjing Medical University, Nanjing, 210009 China; 8 Department of Molecular Pharmacology, Albert Einstein College of Medicine, Bronx, NY 10461 USA; 9 Department of Anatomical, Histological, Forensic Medicine and Orthopedic Science, Sapienza University of Rome, Roma, 5000161 Italia; 10 Department of Anatomical, Histological, Medical and Legal locomotive Apparatus, Section of Human Anatomy Via Alfonso Borelli, Sapienza University of Rome, Roma, 5000161 Italia; 11 Institute for Chemical Carcinogenesis, Guangzhou Medical University, Guangzhou, 511436 China

**Keywords:** Cancer models, Colorectal cancer, Tumour biomarkers, Cancer therapy, Metastasis

## Abstract

Chimeric antigen receptor (CAR) - T cell therapy is a new class of cellular immunotherapies, which has made great achievements in the treatment of malignant tumors. Despite improvements in colorectal cancer (CRC) therapy, treatment of many patients fails because of metastasis and recurrence. The human epidermal growth factor receptor 2 (HER2) is a substantiated target for CAR-T therapy, and has been reported recently to be over-expressed in CRC, which may provide a potential therapeutic target for CRC treatment. Herein, HER2 was a promising target of metastatic colorectal cancer (mCRC) in CAR-T therapy as assessed by flow cytometry and tissue microarray (TMA) with 9-year survival follow-up data. Furthermore, HER2-specific CAR-T cells exhibited strong cytotoxicity and cytokine-secreting ability against CRC cells in vitro. Moreover, through the tumor-bearing model of the NOD-Prkdc^em26cd52^Il2rg^em26Cd22^/Nju (NCG) mice, HER2 CAR-T cells showed signs of effectively preventing CRC progression in three different xenograft models. Notably, HER2 CAR-T cells displayed greater aggressiveness in HER2^+^ CRC in the patient-derived tumor xenograft (PDX) models and had potent immunotherapeutic capacity for mCRC in the metastatic xenograft mouse models. In conclusion, our studies provide scientific evidence that HER2 CAR-T cells represent an emerging immunotherapy for the treatment of mCRC.

## Introduction

Colorectal cancer (CRC) has become a worldwide concern for its severe metastasis and recurrence. About one-third of patients present with metastatic disease at the time of their first diagnosis. The main treatment options are still surgery, chemotherapy, and radiotherapy. It is reported that 40% of the early-stage patients eventually relapse after surgical resection and the 5-year survival rate of patients with advanced disease is only 10–15% [1–3], suggesting that novel therapeutic strategies are urgently needed. At this time, immunotherapy has started to be widely discussed.

Chimeric antigen receptor (CAR)-T immunotherapy is one of the new approaches with superior efficacy for the treatment of malignant tumors, particularly for hematological cancer [4, 5]. CAR-T cells recognize specific antigens in a major histocompatibility (MHC)-independent manner, which leads to the activation and execution of its anti-tumor function [6], targeting and killing cancer cells. Although serious toxicities such as cytokine release syndrome (CRS) and neurological toxicities have been observed, clinical trials have shown that these can be managed with appropriate preparation and training. Currently, the successful application of immunotherapy for solid tumors, such as glioblastoma multiforme and lung cancer, has stimulated the enthusiasm for research on CRC immunotherapy [7]. Notably, in contrast to most other treatments for metastatic cancer, immunotherapy achieves long-term durable remission in a subset of CRC patients, highlighting the tremendous promise of immunotherapy in treating metastatic colorectal cancer (mCRC) [8]. Nevertheless, several obstacles remain to be overcome for the successful application of CAR-T cells in solid tumors. One of the most important goals is to find the specific targetable antigens.

Human epidermal growth factor receptor 2 (HER2) is over-expressed in a range of tumors including breast cancer, gastric cancer, lung cancer, and ovarian cancer, which has been extensively investigated in CAR-T therapy for solid tumors [9–11]. The latest studies have shown that the protein expression of HER2 is increased in CRC as well, thereby suggesting that it can be used as a novel immunotherapy target for CRC [12, 13]. Yet, the specificity and reactivity of HER2 CAR-T cells’ assault on CRC remain to be elucidated. Here, HER2 was identified as a promising target for mCRC in CAR-T therapy by flow cytometry assays and tissue microarray (TMA) with 9-year survival information available from two CRC cohorts. Furthermore, these HER2-specific CAR-T cells exhibited strong anti-CRC effects in a variety of functional assays in vitro and in three different models of xenotransplantation in vivo. Notably, HER2 CAR-T cells displayed greater aggressiveness in the HER2^+^ CRC in patient-derived tumor xenograft (PDX) models and demonstrated potent immunotherapeutic capacity for mCRC in the metastatic xenograft mouse models. Importantly, no adverse effects were observed throughout the research. Overall, these findings provided preclinical evidence for immunotherapeutic targeting of HER2 to treat mCRC.

## Materials and methods

Complete details of all reagents and procedures used in this study are provided in SI Appendix, SI Materials and Methods.

### Specimen collection and annotation

All clinical samples were obtained from patients and healthy volunteers after providing written informed consent. The study was conducted after the approval of the Southeast University Affiliated Zhongda Hospital Ethics Committee.

Two independent cohorts were subjected to the construction of TMAs. A total of 360 CRC patients comprised the Xuzhou cohort that was recruited in The Affiliated Hospital of Xuzhou Medical University, while the Nanjing cohort, consisting of 680 CRC patients, was enrolled from the Jiangsu Tumor Hospital from 2007 to 2011. All patients were newly diagnosed with CRC and histologically confirmed with no preoperative chemo/radiotherapy (Supplementary Table 1). The pathological stage of CRC was assessed using the 7th edition of the American Joint Committee on Cancer Staging Manual. All patients were contacted by follow-up telephone calls until the time of death; to date, the maximum follow-up duration was 112.7 months. Furthermore, a total of 106 pairs of fresh CRC tumors and matched adjacent tissues (5 cm away from the tumoral margins) used for flow cytometry analysis were collected from patients with CRC who underwent surgery at the Jiangsu Tumor Hospital and Jiangsu Province Hospital between 2017 and 2018. The detailed clinical characteristics of patients are listed in Supplementary Table 2.

### Generation of HER2-CAR lentivirus

Second-generation CARs retarget and reprogram the T cells to augment their anti-tumor efficacy [14]. HER2-specific CAR lentivirus was constructed by iCarTab (Suzhou, China) based on the variable region sequences of heavy and light chains of HER2 sub-cloned into the lentivirus expression vector pCAR-puro. After the vector was confirmed by restriction endonucleases digestion and sequencing, the plasmids were extracted and eventually prepared for lentiviral transfection. Subsequently, phosphate-buffered saline (PBS; Gibco) supplemented with 10 μg Lenti-CAR, 11 μg Lenti-Mix, and 26 μL PEI (100 μM; PolyScience, Philadelphia, PA, USA) was added to 6-well cell culture plates (2 mL/well; Corning Incorporated, Corning, NY, USA) and incubated for 10 min at room temperature (RT). Then, the DNA/PEI compound was added to the 15-cm Petri dish (Corning Incorporated) and cultured at 37 °C in 5% CO_2_ for 6 h, followed by inoculating 5 × 10^6^ 293T cells/dish in DMEM complete medium overnight. The medium was refreshed and the culture continued for an additional 42 h. Subsequently, the supernatant was collected by centrifugation at 50,000 × *g* for 2 h, and the pellet resuspended in 500 µL PBS to detect the titer of the lentivirus or stored at −80 °C. As described previously [15, 16], viral titers were measured by quantitative PCR (qPCR; Roche, Basel, Switzerland); the quantification cycle value was defined as the number of cycles when the fluorescent signal reached a specified threshold.

### Recombination of oncolytic adenovirus

The oncolytic adenovirus was created by iCarTab (Suzhou, China) in order to enhance the activity of CAR T cells in CRC. As reported previously [17], the full-length ORF of the target genes *Rantes* and *IL-15* was subcloned into the shuttle plasmid pShuttle-RANTES-IRES-IL-15 (iCarTab) according to the sequences published in GenBank (https://www.ncbi.nlm.nih.gov/genbank/, accession number: NM_002985.2). Furthermore, the adenovirus plasmid pAD-Backbone and the shuttle plasmid vector pShuttle-RANTES-IRES-IL-15 were co-transfected into well-cultured 293A cells using Lipofectamine 2000 (Cat: 11668019, Invitrogen, Carlsbad, CA, USA) to complete the virus recombination. After 96 h incubation at 37 °C in 5% CO_2_, virus plaques were observed and purified three times. Subsequently, 293A cells were alternately frozen and thawed to collect the restructured oncolytic adenovirus, followed by qPCR amplification of AD-RANTES-IL-15 to identify the recombinant oncolytic adenovirus.

### Separation and activation of primary T cells

Peripheral blood mononuclear cells (PBMCs) were isolated from blood samples obtained from healthy donors, aged 20–40 years, recruited from the Southeast University, signed written consent was obtained before the donation of peripheral blood. The blood samples were diluted with PBS plus 2% (v/v) FBS and added to the upper layer of Lymphoprep (Nycomed, Oslo, Norway) before centrifugation at 1006.2 × *g* for 30 min. The mononuclear cell layer at the interface between serum and the separation reagent was collected into 15-mL centrifuge tubes (Corning Incorporated, Corning, NY, USA) and washed with the medium. Furthermore, the cells were counted and maintained in RPMI 1640 medium (Corning Incorporated) in 1.5-mL centrifuge tubes (Corning Incorporated). Dynabeads^TM^ Human T-Expander CD3/CD28 (Cat: 11141D, Gibco, Gaithersburg, MD, USA) was added at a 3:1 bead:T cell ratio at 37 °C in 5% CO_2_ for 1 h. After incubation, the tubes were inserted into the magnetic separation rack at RT for 10 min. The absorbed T cells were resuspended in complete medium supplemented with 10% (v/v) FBS, 100 U/mL penicillin, 100 μg/mL streptomycin, 300 U/mL IL-2 (PeproTech, Rocky Hill, NJ, USA), 10 ng/mL IL-7 (PeproTech), and 5 ng/mL IL-15 (PeproTech).

### Lentivirus infection

After the activated T cells were continuously cultured for 48 h, the T cell density was adjusted to 1 × 10^6^/mL and resuspended in complete RPMI 1640 medium in 6-well plates (1 mL/well). Next, the HER2-specific CAR lentivirus (virus titer: 1 × 10^8^ pfu/mL) was mixed with 6 µg/mL polybrene (Cat: 40804ES76, Yeasen, Shanghai, China), added to the medium and cultured for 6 h at 37 °C in 5% CO_2_; T cells without lentivirus infection were used as the control group (NT-T). The volume of virus that needs to be added was calculated as follows: virus (mL) = (MOI × cell number)/virus titer, according to the multiplicity of infection (MOI) = 15. Finally, the total volume of medium in each well was increased to 2 mL. Moreover, the culture medium containing the virus was replaced with fresh complete medium after 24 h post-infection and cultured for several days to reach the required number of CAR-positive T cells.

### Construction of expression plasmid and lentivirus stable transduction

A lentiviral expression shuttle vector (pLenti6/V5-GW/lacZ; Invitrogen, Carlsbad, CA, USA) that harbored a firefly luciferase cDNA was constructed as described previously [18]. Then, lentiviruses were generated by co-transfection with packaging plasmids pSPAX2 and pMD2G into DLD-1 cells and incubated overnight. After 12 h incubation, the medium was replaced with DMEM complete medium containing 10 μg/mL blasticidin S (InvivoGen, San Diego, CA, USA), and replaced every 2 days for 1 week until all the control cells were excluded. The vector-containing cells were maintained in 1 μg/mL blasticidin S for ~2 weeks, and then, frozen until further use.

### Patient-derived xenograft models

First of all, the fresh CRC tissues were divided into two groups (HER2.High versus HER2.Low) according to the surface expression of HER2 by flow cytometry analysis. Next, the procedures for the development, transplantation, and characterization of tumor grafts generated directly from surgical specimens were similar to those reported previously [19]. The tumor sizes were recorded twice weekly and calculated as follows: V = 0.5 × length × width^2^. When tumors reached a volume of 250 mm^3^, they were excised, cut into multiple fragments (5 × 5 × 5 mm^3^ each), and transplanted into new NOD-Prkdc^em26cd52^Il2rg^em26Cd22^/Nju (NCG) mice to produce the next generation. Moreover, tumors that were not used immediately were frozen in liquid nitrogen. After three passages, single-cell isolation of the PDX tumor cells from xenograft CRC tumor models were obtained as described previously [20].

### Animal treatment

Five-week-old female NCG were purchased from the Model Animal Research Center of Nanjing University (Nanjing, China). All animal experiments were performed according to the guidelines of the Committee on Animal Use and Care of Southeast University. Mice were housed under barrier conditions with 12 h light/dark cycles and ad libitum access to food and water. The mice were randomly divided into each group.

In xenograft models (*n* = 12), each mouse was subcutaneously injected with 5 × 10^6^ DLD-1 cells with the luciferase reporter gene under the armpit (day 1). All mice were injected with the recombinant adenovirus into the tumor by multi-site tumor puncture injection (day 10). Then, 1 × 10^7^ NT-T or HER2 CAR-T cells were injected into a tail vein without exogenous cytokine injection (day 13). Furthermore, the recombinant adenovirus and T cells were injected again on days 18 and 21, respectively. Within this process, all mice were monitored for tumor growth daily and measured twice a week, while the tumor changes were determined on days 7, 17, and 27 by IVIS Spectrum in Vivo Imaging System (IVIS; PerkinElmer/Caliper, Hopkinton, MA, USA). At the end of the experiment, the mice were euthanized and luminescence intensity in tissues (xenograft, lung, liver) was measured by a Chemiluminescence detector (Sirius, Berthold Detection Systems, Germany). The detailed experimental procedures were described previously [21].

Furthermore, in the CRC PDX models (*n* = 24), after three passages, single-cell isolation of the PDX tumor cells (5 × 10^6^/0.2 mL/mouse) was injected subcutaneously into the armpit of NCG mice (day 1). The following procedure was consistent with that of the former xenograft models.

Moreover, in the PDX metastasis models (*n* = 12), single-cell suspension of the PDX tumor cells (1 × 10^6^/0.1 mL/mouse) was injected into NCG mice via a tail vein (day 1). Next, 1 × 10^7^ NT-T or HER2 CAR-T cells were injected on days 7 and 12, respectively. Tumor development in the liver and lungs was monitored by magnetic resonance imaging (MRI) and computed tomography (CT). In addition, at the end of the experiment (4 weeks), all experimental mice were euthanized and hematoxylin and eosin (H&E) staining was used to further validate the distant metastases of the liver and lungs.

## Results

### HER2 is a promising target of mCRC in CAR-T therapy

**Fig. 1:**
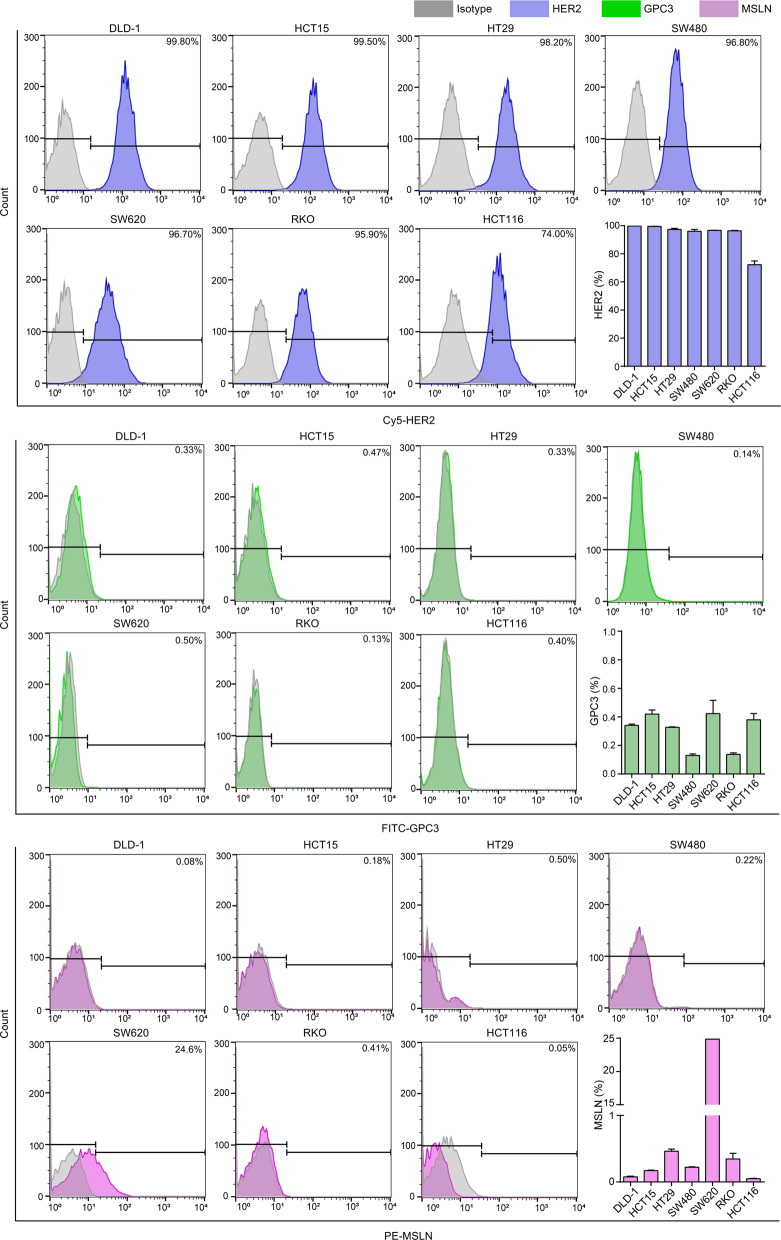
Surface expression levels of three candidate target proteins. Upper: Expression of HER2 in 7 CRC cell lines as detected by flow cytometry. Medial: Expression of GPC3 in 7 CRC cell lines as detected by flow cytometry. Lower: Expression of MSLN in 7 CRC cell lines as detected by flow cytometry.

**Fig. 2:**
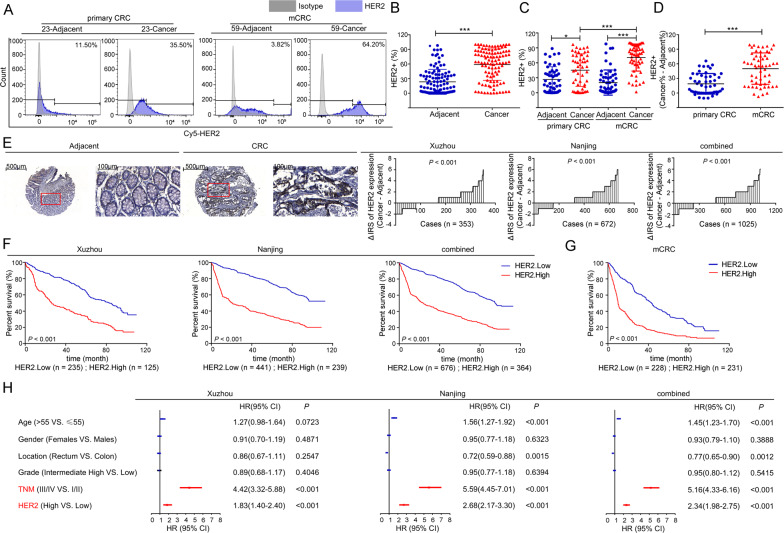
Expression of HER2 in tissues. **A** A total of 106 pairs of fresh CRC tumors and matched adjacent tissues were assessed by flow cytometry analysis, and the representative images are shown. **B** The expression levels of HER2 in fresh tissues of CRC patients (*n* = 106, ^***^P < 0.001, two-tailed *t*-test). **C** The expression levels of HER2 in fresh tissues of CRC patients with different progression. (*P < 0.05, ***P < 0.001, Kruskal–Wallis test followed by the Dunn’s multiple comparison test). **D** The difference in the expression levels of HER2 between fresh CRC tumors and matched adjacent tissues (Cancer% - Adjacent%) of CRC patients with different progression (^***^P < 0.001, two-tailed *t*-test). **E** Left panel: The levels of HER2 in TMA were evaluated by IHC analysis, and the representative staining images are shown. The scale bar is marked in each image. Right panel: The difference in staining score between CRC lesions and adjacent tissues (Cancer - Adjacent) in Xuzhou, Nanjing, and combined cohorts are shown. **F** KM curves depicting the overall survival of CRC patients based upon tumoral HER2 levels. Left to right: Xuzhou, Nanjing, and combined cohorts. **G** KM curves depicting the overall survival of mCRC patients based upon tumoral HER2 levels. *P* values were calculated by the Log-rank test. **H** Multivariable COX regression analyses (adjusted age, gender, location, grade, TNM stage and HER2 expression) were performed in our cohorts. Bars correspond to 95% confidence interval (CI). HR hazard ratio.

Since glypican-3 (GPC3), mesothelin (MSLN), and HER2 are commonly used markers for tumor detection and/or as potential therapeutic targets, and a growing number of studies have confirmed the efficacy of specific CAR-T targeting them has achieved considerable achievements in the immunotherapy of solid tumors [11, 12, 22, 23]. In our study, the surface expression levels of these three candidate target proteins were evaluated in a total of 7 CRC cell lines by flow cytometry. As illustrated in Fig. 1, HER2 was expressed at high levels in all CRC cell lines from 74.00% to 99.80%, but GPC3 and MSLN were rarely expressed. Next, the expression of HER2 protein was detected in 106 pairs of fresh CRC tumors and matched adjacent tissues. The clinical characteristics of these patients are presented in Supplementary Table 2. Consistent with the findings in CRC cells, when compared with matched adjacent tissues, HER2 was expressed at significantly high levels in total, primary (I/II) or metastatic (III/IV) CRC tissues (*P* < 0.001) (Fig. 2A–C and Figs. S1–S5), which was also consistent with the results of others [24, 25]. Of note, the expression level of HER2 in mCRC was particularly high as compared with the other three groups (Fig. 2C), and the difference in the expression level of HER2 between cancer and matched adjacent tissues in mCRC was greater than the difference in primary CRC (Fig. 2D), suggesting that HER2 was a more suitable potential therapeutic target for mCRC.

To further determine whether HER2 was a promising CRC target for CAR-T therapy, protein levels of HER2 were investigated by TMA in two independent cohorts of 1040 CRC patients. Results showed that HER2 was markedly upregulated in tumor tissues than in corresponding adjacent tissues (Fig. 2E), and the higher levels of HER2 were associated with shorter survival (Fig. 2F). In connection with the high HER2 expression levels in mCRC cancer tissues (Fig. 2C), Kaplan–Meier (KM) analysis proved that the prognosis of patients with high levels of HER2 in mCRC patients was worse (Fig. 2G). Moreover, multivariate Cox regression analysis reconfirmed that HER2 was an independent negative prognostic factor for CRC in all cohorts (Fig. 2H and Supplementary Table 3). Collectively, these results demonstrated the value of HER2 as a putative target antigen for the immunotherapy of CRC, especially in patients with mCRC.

### HER2 CAR-T cells possess specific cytotoxic activity against CRC cells

**Fig. 3:**
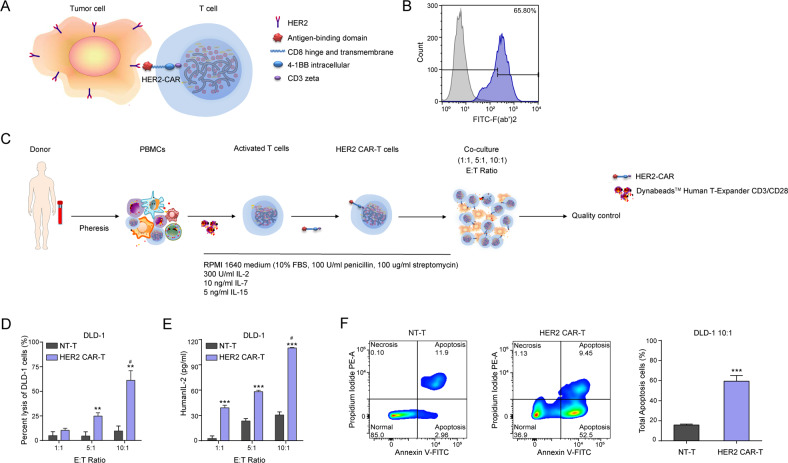
Construction of HER2 CAR-T cells that can specifically target CRC cells. **A** Structure and mechanism of action of HER2 CAR-T. **B** Surface expression of the HER2-CAR on lentivirus infected T cells was detected by flow cytometry on 10th day after lentivirus infection. **C** Procedure of allogeneic HER2 CAR-T therapy. **D**, **E** Cytotoxicity assays and cytokine release assays (*P < 0.05, **P < 0. 01, ***P < 0. 001, compared with NT-T of each sub-group, two-tailed *t*-test; ^#^*P* < 0.05, compared with 1:1 of each sub-group, Kruskal–Wallis test followed by the Dunn’s multiple comparison test). **F** An apoptosis kit was conducted in the co-culture model at the E/T ratio of 10:1, and the representative images of cell apoptosis are shown (***P < 0.001, compared with NT-T, two-tailed *t*-test).

Considering second-generation CARs being superior to first-generation and third-generation in terms of effectiveness [26, 27], a second-generation CAR specifically targeting HER2 was constructed (Figs. 3A and S6). As assessed by flow cytometry, up to the 10th day after lentivirus infection, the percentage of T cells positive for HER2 CAR was still 65.8% (Fig. 3B), suggesting the successful construction of a HER2-specific CAR. As reported, when CAR was specifically bound to the tumor-associated antigens (TAAs), T cells can be activated (cytotoxicity, T cell proliferation, and cytokine production) [28, 29]. In the current study, the specific activity of HER2 CAR-T cells against HER2^+^ tumors was evaluated by incubating the HER2 CAR-T cells with established human CRC cells (DLD-1) at an effector-to-target (E/T) ratio of 1:1, 5:1, and 10:1 in the absence of exogenous cytokines (Fig. 3C). The specific killing activity of HER2 CAR-T cells against DLD-1 cells was significantly higher than NT-T cells according to the lactate dehydrogenase (LDH) assay in a concentration-dependent manner (Fig. 3D). Similarly, the level of interleukin-2 (IL-2) cytokine released by HER2 CAR-T cells was markedly elevated as compared with NT-T cells (Fig. 3E). Simultaneously, the cell apoptosis assay revealed that HER2 CAR-T cells promoted the total apoptosis of DLD-1 cells as compared with the NT-T cells significantly at the E/T ratio of 10:1 (Fig. 3F). Hence, we confirmed that HER2 CAR-T cells possessed potent and specific cytotoxic activity against CRC cells.

### HER2 CAR-T cells exhibit antitumor activity in CRC xenograft models

**Fig. 4:**
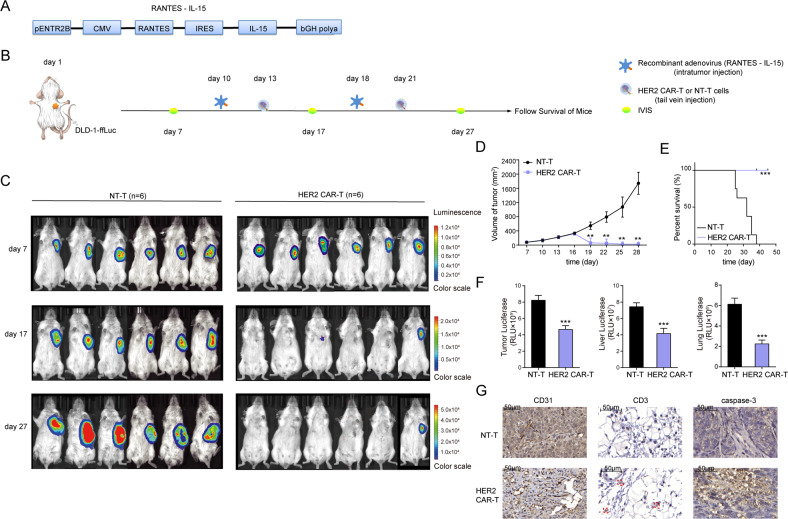
HER2 CAR-T cells exhibit antitumor activity in CRC xenograft models. **A** Structure of recombinant adenovirus overexpressing RANTES-IL-15. **B** Schematic of the xenograft models (*n* = 12). NCG mice were injected subcutaneously with 5 × 10^6^ DLD-1-ffLuc on day 1, followed by injection of recombinant adenovirus on days 10 and 18. HER2 CAR-T or NT-T cells (1 × 10^7^) were injected via tail vein on days 13 and 21, respectively. **C** IVIS followed by DLD-1-ffLuc injection, on days 7, 17, and 27, and the representative images are shown. **D** The volume of tumors was measured twice a week (**P < 0.01, compared with NT-T, two-tailed *t*-test). **E** KM analysis of the overall survival of mice. (***P < 0.001, compared with NT-T, Log-rank test). **F** Flank tumor, liver, and lung metastatic burden in mice (***P < 0.001, compared with NT-T of each sub-group, two-tailed *t*-test). **G** The protein levels of CD31, CD3, and caspase-3 in mice were evaluated by IHC, and the representative images are shown. The scale bar is marked in each image.

We then assessed the anti-tumor activity of the HER2 CAR-T cells in vivo. Firstly, recombinant adenovirus over-expressing RANTES-IL-15 was constructed to promote T-cell proliferation and migration (Fig. 4A) [30]. Next, a xenogeneic model of CRC expressing the firefly luciferase reporter gene (DLD-1-ffLuc) in NCG mice was utilized, and the detailed construction process was shown in Fig. 4B. According to the IVIS Spectrum in Vivo Imaging System (IVIS), tumors in HER2 CAR-T cells-treated mice were significantly controlled, among which, tumors of 4 mice were even eliminated since day17, while tumors in NT-T cells-treated mice continued to grow rapidly without any anti-tumor effects (Fig. 4C, D). Correspondingly, mice in the HER2 CAR-T group exhibited significantly enhanced overall survival time, relative to those in the NT-T group (Fig. 4E). Moreover, luciferase biochemical measurements data demonstrated that the tumor progression in the HER2 CAR-T group was suppressed, as well as the distant metastases to liver and lungs (Fig. 4F). Importantly, no adverse effects were observed during the in vivo assay, confirming the safety of HER2 CAR-T therapy for clinical application.

To assess the homing ability of HER2 CAR-T cells, protein levels of CD31, CD3, and caspase-3 were evaluated on tumor samples from euthanized mice by immunohistochemistry (IHC) staining. The results showed considerable increases in the recruitment of human CD3-positive T cells and higher caspase-3 expression levels, and decreased CD31 levels in the HER2 CAR-T group, indicating decreased angiogenesis, enhanced T-cell activation, and aggravated cell apoptosis, respectively (Fig. 4G). In sum, these findings strongly confirmed that HER2 CAR-T cells could effectively traffic to target sites and had strong anti-tumor properties for CRC xenograft models.

### HER2 CAR-T cells display greater aggressiveness in HER2^+^ CRC in PDX models

**Fig. 5:**
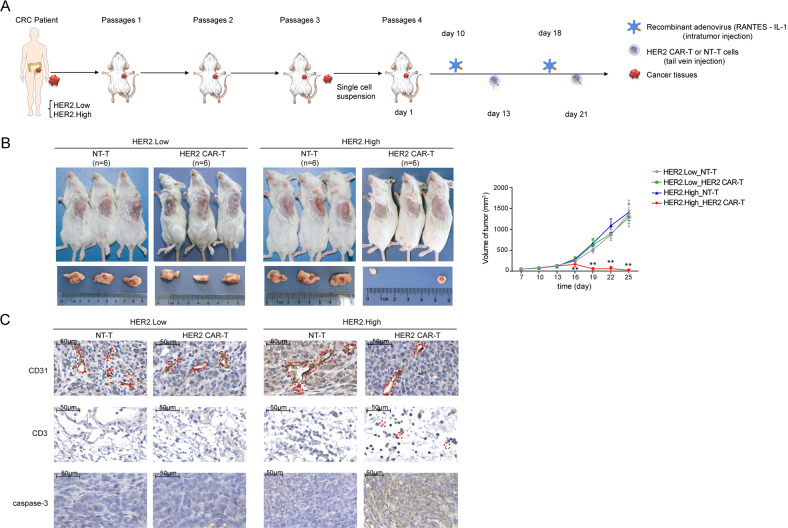
HER2 CAR-T cells display greater aggressiveness in HER2^+^ CRC in PDX models. **A** Left panel: Fresh human CRC tissues were transplanted into NCG mice. After three passages, a singlecell suspension of the PDX tumor cells (HER2.Low: low expression of HER2, HER2.High: high expression of HER2) was obtained. Right panel: Schematic of the PDX models (*n* = 24). NCG mice were injected subcutaneously with single-cell suspension on day 1, and recombinant adenovirus was injected on days 10 and 18. HER2 CAR-T or NT-T cells (1 × 10^7^) were injected via a tail vein on days 13 and 21, respectively. **B** The volume of the tumor was measured twice a week (**P < 0.01, HER2 CAR-T compared with NT-T in HER2.High group, two-tailed *t*-test). **C** The protein levels of CD31, CD3, and caspase-3 in mice were evaluated by IHC, and the representative images are shown. The scale bar is marked in each image.

PDX models mimic the microenvironment of the tumor of origin and retain the morphological and genomic features of their original counterparts, linking the responses in the xenografts and predictability responses seen in patients [31, 32], and might serve as an attractive alternative to generate and evaluate the efficacy of CAR-T-cell products. In this study, CRC PDX models were utilized to further evaluate the anti-tumor effects of HER2 CAR-T cells [33]. Considering tumor heterogeneity, CRC PDX models using the specimens obtained from CRC patients with different HER2 expression levels (HER2.High versus HER2.Low) were successfully established and characterized (Supplementary Table 4). After three passages, single cells isolated from the PDX tumor were injected subcutaneously into the armpit of NCG mice. The following procedure was consistent with that of the xenograft models (Fig. 5A). In the HER2.High group, mice treated with HER2 CAR-T cells exhibited considerably decreased tumor size as compared with the NT-T cells, while subtle changes in the HER2.Low group (Fig. 5B), suggesting that HER2 CAR-T cells effectively infiltrated into tumor tissues and selectively eliminated HER2^+^ CRC cells, implying the excellent therapeutic effect of HER2 CAR-T cells on HER2^+^ CRC. In addition, consistent results were obtained by IHC assay. When compared with the NT-T cells, HER2 CAR-T cells markedly enhanced T-cell activation, inhibited angiogenesis, and promoted cell apoptosis in the HER2.High group, however, no remarkable changes were observed in the HER2.Low group (Fig. 5C). Taken together, it could be concluded that HER2 CAR-T cells were aggressive against CRC when HER2 was elevated.

### HER2 CAR-T cells have effective immunotherapeutic capacity for mCRC

**Fig. 6:**
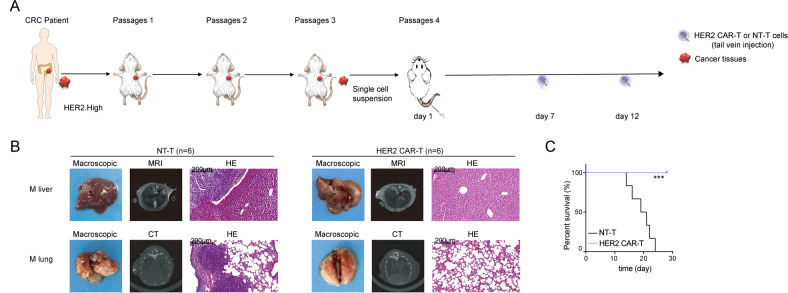
HER2 CAR-T cells have effective immunotherapeutic capacity for mCRC. **A** Left panel: Fresh human CRC tissues were transplanted into NCG mice. After three passages, single-cell suspension of the PDX tumor cells (HER2.High: high expression of HER2) was obtained. Right panel: Schematic of the PDX metastasis models (*n* = 12). Then, two consecutive injections of T cells (1 × 10^7^) were administered after single-cell suspension was injected into the NCG mice via a tail vein. **B** MRI, CT, and H&E were performed to evaluate the pathological changes in the liver and lungs (M liver: liver metastasis, M lung: lung metastasis), and the representative images are shown. The scale bar is marked in each image. **C** KM analysis of the overall survival of mice. (***P < 0.001, compared with NT-T, Log-rank test).

Since HER2 exhibited significantly increased levels in mCRC as compared with primary CRC (Fig. 2C, D), CRC PDX models showed that HER2 CAR-T displayed great aggressiveness in HER2^+^ CRC (Fig. 5), and HER2 CAR-T cells were associated with the suppression of distant metastases in CRC (Fig. 4F) in order to investigate the therapeutic capacity of HER2 CAR-T cells for mCRC, a PDX metastasis model of CRC was developed. As depicted in Fig. 6A, two consecutive injections of T cells were administered, after single-cell isolation of the CRC PDX tumor cells with high HER2 levels was injected into NCG mice via tail vein. Compared with the NT-T group, magnetic resonance imaging (MRI) and computed tomography (CT) images showed that the distant metastases were significantly inhibited in the HER2 CAR-T group. Similar results were observed by Hematoxylin and Eosin (H&E) staining (Fig. 6B). Furthermore, all mice in the HER2 CAR-T group survived to the end of the experiment (Fig. 6C). The current study therefore strongly confirmed the effective immunotherapeutic capacity of HER2 CAR-T cells for mCRC.

### Safety of HER2 CAR-T cells

**Fig. 7:**
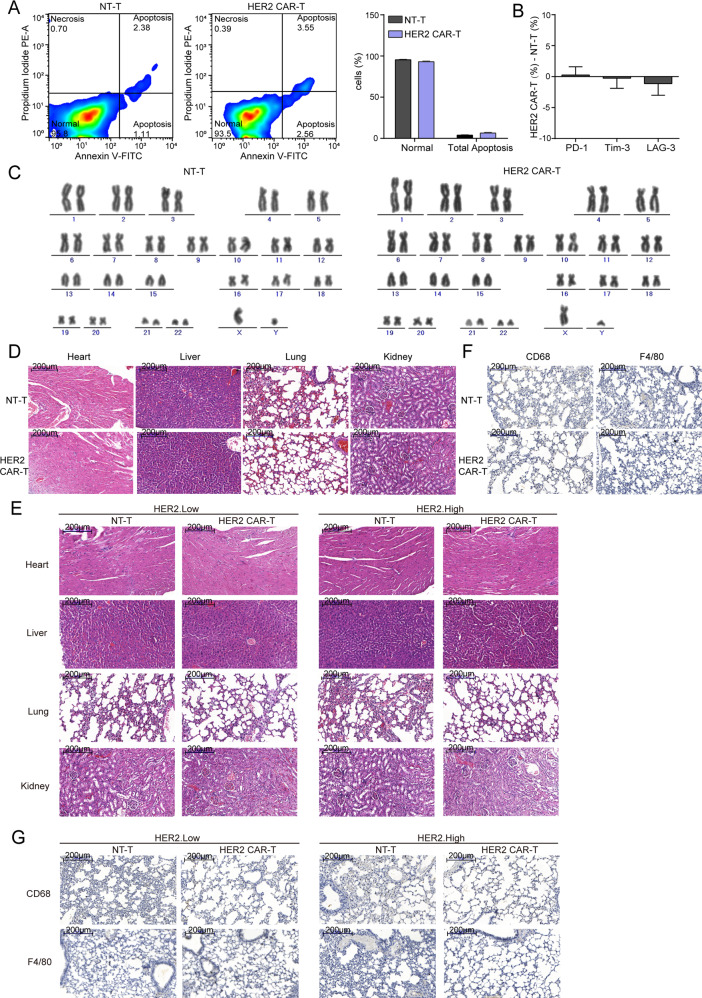
Safety of HER2 CAR-T cells. **A** 7 days after lentivirus infection, an apoptosis detection kit was used to detect the apoptosis of T cells, and the representative images are shown. **B** The difference in the expression levels of three exhaustion markers between HER2 CAR-T and NT-T group (HER2 CAR-T (%) - NT-T (%)) as detected by flow cytometry on 7 days after lentivirus infection. **C** 14 days after lentivirus infection, karyotype analysis was performed to detect chromosomal alterations in CAR-T cells. **D**, **E** H&E was performed to evaluate the pathological changes in Heart, Liver, Lung, and Kidney, and the representative images are shown. The scale bar is marked in each image. **F**, **G** IHC assay was performed to observe F4/80 or CD 68 positive macrophages in murine lung tissues, and the representative images are shown. The scale bar is marked in each image.

Given lentivirus-mediated gene transfer might lead to cell genome instability and produce certain toxicity, apoptosis assay, and karyotype analysis were performed to evaluate the safety of HER2-CAR expression by lentivirus infection. Firstly, apoptosis assay showed that on the 7th day after lentivirus infection, the proportion of total apoptosis cells in both groups was very low, and a large proportion of them were still normal living cells (Fig. 7A). Meanwhile, flow cytometry analysis showed that the expression levels of the exhaustion markers (PD-1^+^, LAG3^+^, and Tim3^+^) in the HER2 CAR-T group were not significantly different from that in the NT-T group (Fig. 7B). Furthermore, HER2 CAR-T cells maintained a normal karyotype even after 14-day lentivirus infection (Fig. 7C). Moreover, pathological inspections of vital organs were performed to investigate pathological changes by H&E staining in CRC xenotransplantation models. Results showed that either in CRC xenograft models or PDX models, no obvious pathological changes were found in heart, liver, lung, and kidney sections in any of the treatment groups (Fig. 7D, E). In addition, CD68^+^ or F4/80^+^ (markers of macrophage) cells were rarely to be observed in the lung tissues from HER2 CAR-T treatment groups (Fig. 7F, G). In combination with the previous safety targeting CRC effect of HER2 CAR-T both in vivo and in vitro, we might draw a conclusion that HER2 CAR-T cells represent an emerging immunotherapy for the treatment of mCRC.

## Discussion

CAR-T therapy has achieved remarkable success in a variety of relapsed/refractory malignant diseases, especially for hematological cancer [34–36], which can specifically localize and eliminate tumor cells by interacting with the TAAs expressed on the tumor cell surface [28]. Recently, additional studies proposed the use of CAR-T therapy for solid tumors [28, 37]; however, whether patients with solid tumors would receive the same benefits as patients with liquid tumors remains uncertain, and the therapeutic application against solid tumors faces major challenges largely owing to the dense extracellular matrix, the complex heterogeneity and immunosuppressive microenvironment of solid tumors [33]. Encouragingly, recent studies demonstrated that while CRS can be devastating during the treatment of liquid tumors, the risk for “on-target/on-tumor” toxicity for solid tumors seems to be lower [38]. This is sufficient to introduce CAR-T therapy into the treatment of solid tumors.

As an immunogenic tumor, CRC has gradually attracted widespread attention. Immunotherapy can avoid the shortcomings of existing drug treatments, such as drug resistance, poor efficacy and serious toxicities, and provide new strategies for the patients with relapsed or metastatic CRCs, who have a poor response to chemo- and/or radiotherapy. Currently, CAR-T therapy for CRC is still in its infancy. A key factor responsible for the poor specificity and efficacy of CAR-T therapy against malignant cells is the lack of specific targetable antigens [38]; therefore, finding a specific target antigen is crucial for CAR-T therapy in the treatment of CRCs.

A large number of CAR-T therapeutic targets have emerged during the rapid development of CAR-T therapy for solid tumors, and some have been used in clinical trials. For instance, anti-epidermal growth factor receptor (EGFR) and EGFR variants have been developed for treating glioma [39, 40]. CARs specific to GPC3 are undergoing clinical evaluation in patients with hepatocellular carcinoma and lung squamous cell carcinoma [41, 42]. In addition, MSLN-specific CAR-T cells in patients with malignant tumors have shown anti-tumor activity [43, 44]. HER2-targeted CAR-T cells effectively eliminated numerous HER2^+^ solid tumors [45, 46]. According to the maturity of clinical trials and the diversity of expression distribution in different malignancies, the surface expression of GPC3, MSLN, and HER2 on CRC cell lines was evaluated in this research, which demonstrated that HER2 is a promising target of mCRC for CAR-T therapy, consistent with the results of flow cytometry and TMA, similar to that proposed by Greally et al. [12, 13, 33]. Notably, a clinical trial conducted at NCI to test third-generation HER2 CAR-T cells (CD28/4-1BB/CD3ζ) in patients with metastatic cancers (NCT00924287); however, the trial was terminated after a patient with CRC died of severe acute respiratory failure as a result of the treatment. Meanwhile, in a clinical trial at Baylor, patients with advanced-stage sarcoma were treated with second-generation HER2 CAR-T cells (CD28/CD3ζ) (NCT00902044). It reported that the first 19 patients did not have adverse side effects related to T-cell infusion [45]. Some scholars suggested that the discrepancies in safety between the NCI and Baylor might be due to the different sources of ScFV used, different pretreatment patients received, as well as the different doses of cells and cytokines [23]. Hereby, HER2 CAR-T cells require further preclinical development and testing to improve clinical safety and effectiveness.

In this present study, a second-generation CAR was constructed, which was confirmed to be superior to first-generation and third-generation in terms of effectiveness [27]. Furthermore, the specificity and reactivity of HER2 CAR-T cells against CRC were validated both in vitro and in vivo. Importantly, in the in vitro model, when the T cells were infected with the HER2-CAR lentivirus for 7 days, the apoptosis assay confirmed that HER2-CAR did not have any side effects on the activity of T cells. Meanwhile, the exhaustion state of HER2 CAR-T and NT-T cells was similar. Moreover, HER2 CAR-T cells maintained a normal karyotype even at 14 days after infection with HER2-CAR lentivirus. In addition, throughout the experiments of the three CRC xenotransplantation models, no toxicities were observed in the HER2 CAR-T group, and the overall survival time of HER2 CAR-T cells-treated mice was significantly prolonged, confirming the powerful anti-tumor effect of HER2 CAR-T on CRC and the safety of its clinical application.

Nevertheless, clinical trials are full of uncertainty and therapeutic strategies need to be revised with the times. In order to reduce the off-target toxicity of CAR-T cells, some studies suggest the construction of switchable CAR-T cells that might offer the potential for safety without compromising efficacy due to its titratability [47]; or creating dual-targeted CAR-T cells [23], which might be taken into account in future research. Furthermore, to improve the antitumor efficacy and persistence, “armored” CARs were essential to secrete pro-inflammatory cytokines to protect the CAR-T cells from the inhibitory tumor microenvironment. In addition, the programmed cell death protein 1 (PD-1) dominant-negative receptor-expressing gene was added to CAR-T cells to provide a cell-intrinsic checkpoint blockade [48]. Moreover, because of the genetic heterogeneity, personalized modification during CAR construction might be required for a maximum antitumor effect [37]. As most of the anti-tumor treatments have significant toxicities, occasional adverse events are also inevitable in CAR-T therapy. In addition to the CRS mentioned earlier, another major toxicity is CAR-T cell-related encephalopathy syndrome (CRES), which could be mild and self-limiting when occurring during or shortly after CRS resolution. More and more studies are paying attention to this problem, and they are exploring how to effectively avoid these toxicities and have made some significant progress [49]. Safety and efficacy are two important principles that need to be considered throughout the CAR-T research.

Some of the latest research on CAR-T immunotherapy carry out either mice re-expose/re-challenge models to detect the immune memory capacity of CAR-T therapy [50], or a single injection of CAR-T cells which is sufficient to achieve effective immune capacity for cancer therapy [51]. These two models should be investigated in our further study.

Given the prior experience and future possibilities, the introduction of CAR-T therapy into the treatment of solid tumors was boldly explored. To the best of the author’s knowledge, this is the first study to confirm that HER2 CAR-T is an emerging immunotherapy for mCRC through a series of rigorous experiments both in vitro and in vivo, providing a novel treatment strategy for the recovery of these patients.

## Supplementary information

Supplementary documents

Supplementary figures

## Data Availability

All data relevant to the study are included in the article or uploaded as supplementary information.

## References

[1] Arnold M, Sierra MS, Laversanne M, Soerjomataram I, Jemal A, Bray F (2017). Global patterns and trends in colorectal cancer incidence and mortality. Gut.

[2] Chand M, Keller DS, Mirnezami R, Bullock M, Bhangu A, Moran B (2018). Novel biomarkers for patient stratification in colorectal cancer: a review of definitions, emerging concepts, and data. World J Gastrointest Oncol.

[3] Torre LA, Bray F, Siegel RL, Ferlay J, Lortet-Tieulent J, Jemal A (2015). Global cancer statistics, 2012. CA Cancer J Clin.

[4] Ghosh A, Smith M, James SE, Davila ML, Velardi E, Argyropoulos KV (2017). Donor CD19 CAR T cells exert potent graft-versus-lymphoma activity with diminished graft-versus-host activity. Nat Med.

[5] Ramos CA, Grover NS, Beaven AW, Lulla PD, Wu MF, Ivanova A, et al. Anti-CD30 CAR-T cell therapy in relapsed and refractory hodgkin lymphoma. J Clin Oncol. 2020; JCO2001342. 10.1200/JCO.20.01342 PMC7655020 32701411

[6] Cartellieri M, Bachmann M, Feldmann A, Bippes C, Stamova S, Wehner R (2010). Chimeric antigen receptor-engineered T cells for immunotherapy of cancer. J Biomed Biotechnol.

[7] Hu Z, Zheng X, Jiao D, Zhou Y, Sun R, Wang B (2020). LunX-CAR T cells as a targeted therapy for non-small cell lung cancer. Mol Ther Oncolytics.

[8] Ganesh K, Stadler ZK, Cercek A, Mendelsohn RB, Shia J, Segal NH (2019). Immunotherapy in colorectal cancer: rationale, challenges and potential. Nat Rev Gastroenterol Hepatol.

[9] Slamon DJ, Leyland-Jones B, Shak S, Fuchs H, Paton V, Bajamonde A (2001). Use of chemotherapy plus a monoclonal antibody against HER2 for metastatic breast cancer that overexpresses HER2. N. Engl J Med.

[10] Hudis CA (2007). Trastuzumab-mechanism of action and use in clinical practice. N. Engl J Med.

[11] Song Y, Tong C, Wang Y, Gao Y, Dai H, Guo Y (2018). Effective and persistent antitumor activity of HER2-directed CAR-T cells against gastric cancer cells in vitro and xenotransplanted tumors in vivo. Protein Cell.

[12] Greally M, Kelly CM, Cercek A (2018). HER2: an emerging target in colorectal cancer. Curr Probl Cancer.

[13] La Salvia A, Lopez-Gomez V, Garcia-Carbonero R (2019). HER2-targeted therapy: an emerging strategy in advanced colorectal cancer. Expert Opin Investig Drugs.

[14] Ferreri AJ, Illerhaus G, Zucca E, Cavalli F, International Extranodal Lymphoma Study G. Flows and flaws in primary central nervous system lymphoma. Nat Rev Clin Oncol*.* 2010;7: doi:10 1038/nrclinonc 2010 1039-c1031; author reply doi:1010:1038/nrclinonc 2010 1039-c1032. 10.1038/nrclinonc.2010.9-c1 20700952

[15] Luo M, Huang H, Hou L, Shao S, Huang S, Zhao X (2017). Construction and expression of a lentivirus expression vector carrying the VEGF165-EGFP fusion gene in breast cancer MCF-7 cells. Oncol Lett.

[16] Wang S, Zeng X, Liu Y, Liang C, Zhang H, Liu C (2012). Construction and characterization of a PDCD5 recombinant lentivirus vector and its expression in tumor cells. Oncol Rep..

[17] Liu F, Xu K, Yang H, Li Y, Liu J, Wang J (2018). A novel approach to glioma therapy using an oncolytic adenovirus with two specific promoters. Oncol Lett.

[18] Li X, Lv Y, Gao N, Sun H, Lu R, Yang H (2016). microRNA-802/Rnd3 pathway imposes on carcinogenesis and metastasis of fine particulate matter exposure. Oncotarget.

[19] Garcia PL, Council LN, Christein JD, Arnoletti JP, Heslin MJ, Gamblin TL (2013). Development and histopathological characterization of tumorgraft models of pancreatic ductal adenocarcinoma. PLoS ONE.

[20] Qu L, Ding J, Chen C, Wu ZJ, Liu B, Gao Y (2016). Exosome-transmitted lncARSR promotes sunitinib resistance in renal cancer by acting as a competing endogenous RNA. Cancer Cell.

[21] Abdelwahab MG, Sankar T, Preul MC, Scheck AC (2011). Intracranial implantation with subsequent 3D in vivo bioluminescent imaging of murine gliomas. J Visualized Exp.

[22] Slamon DJ, Clark GM, Wong SG, Levin WJ, Ullrich A, McGuire WL (1987). Human breast cancer: correlation of relapse and survival with amplification of the HER-2/neu oncogene. Science.

[23] Jackson HJ, Rafiq S, Brentjens RJ (2016). Driving CAR T-cells forward. Nat Rev Clin Oncol.

[24] Taieb J, Jung A, Sartore-Bianchi A, Peeters M, Seligmann J, Zaanan A (2019). The evolving biomarker landscape for treatment selection in metastatic colorectal cancer. Drugs.

[25] Siena S, Sartore-Bianchi A, Marsoni S, Hurwitz HI, McCall SJ, Penault-Llorca F (2018). Targeting the human epidermal growth factor receptor 2 (HER2) oncogene in colorectal cancer. Ann Oncol.

[26] Haso W, Lee DW, Shah NN, Stetler-Stevenson M, Yuan CM, Pastan IH (2013). Anti-CD22-chimeric antigen receptors targeting B-cell precursor acute lymphoblastic leukemia. Blood.

[27] Ramello MC, Benzaïd I, Kuenzi BM, Lienlaf-Moreno M, Kandell WM, Santiago DN (2019). An immunoproteomic approach to characterize the CAR interactome and signalosome. Sci Signal.

[28] Yu S, Li A, Liu Q, Li T, Yuan X, Han X (2017). Chimeric antigen receptor T cells: a novel therapy for solid tumors. J Hematol Oncol.

[29] Kershaw MH, Westwood JA, Slaney CY, Darcy PK (2014). Clinical application of genetically modified T cells in cancer therapy. Clin Transl Immunol.

[30] Shang Y, Chai N, Gu Y, Ding L, Yang Y, Zhou J (2014). Systematic immunohistochemical analysis of the expression of CD46, CD55, and CD59 in colon cancer. Arch Pathol Lab Med.

[31] Zhang X, Lewis MT (2013). Establishment of patient-derived xenograft (PDX) models of human breast cancer. Curr Protoc Mouse Biol.

[32] Bertotti A, Migliardi G, Galimi F, Sassi F, Torti D, Isella C (2011). A molecularly annotated platform of patient-derived xenografts (“xenopatients”) identifies HER2 as an effective therapeutic target in cetuximab-resistant colorectal cancer. Cancer Discov.

[33] Teng R, Zhao J, Zhao Y, Gao J, Li H, Zhou S (2019). Chimeric antigen receptor-modified T cells repressed solid tumors and their relapse in an established patient-derived colon carcinoma xenograft model. J Immunother.

[34] Wang J, Chen S, Xiao W, Li W, Wang L, Yang S (2018). CAR-T cells targeting CLL-1 as an approach to treat acute myeloid leukemia. J Hematol Oncol.

[35] Hiramatsu H (2018). Chimeric antigen receptor T cell (CAR-T) therapy for refractory/relapsed hematological malignancy. [Rinsho ketsueki] Jpn J Clin Hematol.

[36] Kasakovski D, Xu L, Li Y (2018). T cell senescence and CAR-T cell exhaustion in hematological malignancies. J Hematol Oncol.

[37] Li J, Li W, Huang K, Zhang Y, Kupfer G, Zhao Q (2018). Chimeric antigen receptor T cell (CAR-T) immunotherapy for solid tumors: lessons learned and strategies for moving forward. J Hematol Oncol.

[38] D’Aloia MM, Zizzari IG, Sacchetti B, Pierelli L, Alimandi M (2018). CAR-T cells: the long and winding road to solid tumors. Cell death Dis.

[39] O’Rourke DM, Nasrallah MP, Desai A, Melenhorst JJ, Mansfield K, Morrissette JJD (2017). A single dose of peripherally infused EGFRvIII-directed CAR T cells mediates antigen loss and induces adaptive resistance in patients with recurrent glioblastoma. Sci Transl Med.

[40] Johnson LA, Scholler J, Ohkuri T, Kosaka A, Patel PR, McGettigan SE (2015). Rational development and characterization of humanized anti-EGFR variant III chimeric antigen receptor T cells for glioblastoma. Sci Transl Med.

[41] Li K, Pan X, Bi Y, Xu W, Chen C, Gao H (2016). Adoptive immunotherapy using T lymphocytes redirected to glypican-3 for the treatment of lung squamous cell carcinoma. Oncotarget.

[42] Shi D, Shi Y, Kaseb AO, Qi X, Zhang Y, Chi J (2020). Chimeric antigen receptor-glypican-3 T-cell therapy for advanced hepatocellular carcinoma: results of phase I trials. Clin Cancer Res.

[43] Beatty GL, Haas AR, Maus MV, Torigian DA, Soulen MC, Plesa G (2014). Mesothelin-specific chimeric antigen receptor mRNA-engineered T cells induce anti-tumor activity in solid malignancies. Cancer Immunol Res.

[44] Maus MV, Haas AR, Beatty GL, Albelda SM, Levine BL, Liu X (2013). T cells expressing chimeric antigen receptors can cause anaphylaxis in humans. Cancer Immunol Res.

[45] Ahmed N, Brawley VS, Hegde M, Robertson C, Ghazi A, Gerken C (2015). Human epidermal growth factor receptor 2 (HER2) -specific chimeric antigen receptor-modified T cells for the immunotherapy of HER2-positive sarcoma. J Clin Oncol.

[46] Morgan RA, Yang JC, Kitano M, Dudley ME, Laurencot CM, Rosenberg SA (2010). Case report of a serious adverse event following the administration of T cells transduced with a chimeric antigen receptor recognizing ERBB2. Mol Ther.

[47] Raj D, Yang MH, Rodgers D, Hampton EN, Begum J, Mustafa A (2019). Switchable CAR-T cells mediate remission in metastatic pancreatic ductal adenocarcinoma. Gut.

[48] Chen N, Morello A, Tano Z, Adusumilli PS (2017). CAR T-cell intrinsic PD-1 checkpoint blockade: a two-in-one approach for solid tumor immunotherapy. Oncoimmunology.

[49] Staedtke V, Bai RY, Kim K, Darvas M, Davila ML, Riggins GJ (2018). Disruption of a self-amplifying catecholamine loop reduces cytokine release syndrome. Nature.

[50] Du H, Hirabayashi K, Ahn S, Kren NP, Montgomery SA, Wang X (2019). Antitumor responses in the absence of toxicity in solid tumors by targeting B7-H3 via chimeric antigen receptor T cells. Cancer Cell.

[51] Zhou S, Li W, Xiao Y, Zhu X, Zhong Z, Li Q (2021). A novel chimeric antigen receptor redirecting T-cell specificity towards CD26(+) cancer cells. Leukemia.

