# Supplementary material for: HER2-specific chimeric antigen receptor-T cells for targeted therapy of metastatic colorectal cancer
Source: Cell Death Dis. 2021 Nov 27;12(12):1109. doi: 10.1038/s41419-021-04100-0 (PMC8627513; doi:10.1038/s41419-021-04100-0)
Supplement: Supplementary file 1 — Supplementary documents [file 41419_2021_4100_MOESM1_ESM.docx]

**HER2-specific Chimeric Antigen Receptor-T Cells for Targeted Therapy of Metastatic Colorectal Cancer**

**Running title: HER2, A Promising Target in Metastatic Colorectal Cancer**

Jie Xu^1,^ ^2^, Qingtao Meng^3,^ ^4^, Hao Sun^2^, Xinwei Zhang^5^, Jun Yun^2^, Bin Li^2^, Shenshen Wu^3,^ ^4^, Xiaobo Li^2,^ ^3,^ ^4^, Hongbao Yang^6^, Haitao Zhu^7^, Michael Aschner^8^, Michela Relucenti^9^, Giuseppe Familiari^10^, Rui Chen^2,^ ^3,^ ^4,^ ^11§^

^1^ School of Public Health, Kunming Medical University, Kunming 650500, China

^2^ Key Laboratory of Environmental Medicine Engineering, Ministry of Education, School of Public Health, Southeast University, Nanjing 210009, China

^3^ School of Public Health, Advanced Innovation Center for Human Brain Protection, Capital Medical University, Beijing 100069, China

^4^ Beijing Key Laboratory of Environmental Toxicology, Capital Medical University, Beijing 100069, China

^5^ Nanjing Municipal Center for Disease Control and Prevention, Nanjing 210003, China

^6^ Center for Drug Safety Evaluation and Research, China Pharmaceutical University, Nanjing 211198, China

^7^ Colorectal Cancer Center, Department of General Surgery, Jiangsu Cancer Hospital, Cancer Research Institute, Cancer hospital of Nanjing Medical University, Nanjing 210009, China

^8^ Department of Molecular Pharmacology, Albert Einstein College of Medicine, NY 10461, USA

^9^ Department of Anatomical, Histological, Forensic Medicine and Orthopedic Science, Sapienza University of Rome, Roma 5000161, Italia

^10^ Department of Anatomical, Histological, Medical and Legal locomotive Apparatus, Section of Human Anatomy Via Alfonso Borelli, Sapienza University of Rome, Roma 5000161, Italia

^11^ Institute for Chemical Carcinogenesis, Guangzhou Medical University, Guangzhou 511436, China

^§^ **Correspondence author: Rui Chen**, Key Laboratory of Environmental Medicine Engineering, Ministry of Education, School of Public Health, Southeast University, 87 Dingjiaqiao, Gulou District, Nanjing 210009, China. Tel: +86 25 83272560; Fax: +86 25 83324322; Email: [101011816@seu.edu.cn](mailto:101011816@seu.edu.cn).

**SI Materials and Methods**

**Cell lines**

CRC cell lines DLD-1, HT29, SW620, HCT15, SW480, HCT116, and RKO were obtained from American Type Culture Collection (ATCC; Manassas, VA, USA) in 2016. These cells were tested by short tandem repeat analysis, validated to be free of mycoplasma and used within 6 months. In addition, the cells were cultured within 25 passages for all experiments. All CRC cell lines were maintained in Dulbecco’s modified Eagle’s medium (DMEM; Gibco, Gaithersburg, MD, USA) supplemented with 10% (v/v) foetal bovine serum (FBS; Sigma, St. Louis, MO, USA), 100 U/mL penicillin (Gibco), and 100 μg/mL streptomycin (Gibco) at 37 °C in 5% CO_2_.

**Tissue microarray and immunohistochemistry staining**

The tissue microarrays (TMAs) were constructed as reported previously ^1^; these TMAs were created by the National Engineering Center for Biochip (Shanghai, China). Freshly cut TMA sections were analyzed by incubating them with anti-HER2 (Cat: ab245702, Abcam, Cambridge, MA, USA) antibodies overnight at 4 °C. Immunohistochemistry (IHC) staining was performed as described previously ^2^. Sections from xenografts or organs were incubated with anti-caspase-3 (Cat: ab13847, Abcam), anti-CD31 (Cat: ab28364, Abcam), anti-CD3 (Cat: ab16669, Abcam), anti-CD68 (Cat: ab955, Abcam) and F4/80 (Cat: 70076, Cell Signaling Technology, Danvers, MA, USA) antibodies. Each section was independently examined by two experienced histologists using microscopy in a blinded manner. A semi-quantitative immunoreactivity score (IRS) as reported ^3^ was used to evaluate the expression levels of proteins on tissues.

**Flow cytometry analysis**

A BD FACS Calibur flow cytometer (BD Biosciences, Franklin Lakes, NJ, USA) was used in this study. In order to estimate the expression levels of HER2, glypican 3 (GPC3), and mesolithin (MSLN) on the cell surface, CRC cells were analyzed by flow cytometry. Briefly, the cells were incubated with anti-human HER2/Neu (Cat: 745890, BD Biosciences), anti-human glypican 3 (Cat: 100393-R024, Sino Biological, Beijing, China), and anti-human mesothelin APC‑conjugated monoclonal (Cat: FAB32652A, R&D Systems, Emeryville, CA, USA) antibodies according to the manufacturer’s instructions. Furthermore, the transduction efficiency of HER2-CAR in T cells was detected by anti-human IgG-F(ab')2 fragment antibody (Cat: A80-149F, BETHYL, Montgomery, TX, USA). In addition, HER2 CAR-T cells and NT-T cells were incubated with anti-human CD223 (LAG-3) (Cat: 369206, BioLegend, San Diego, CA, USA), anti-human CD336 (Tim-3) (Cat: 345005, BioLegend), and anti-human CD279 (PD-1) (Cat: 329905, BioLegend) antibodies to detect the exhaustion state of T cells. A total of 1 × 10^6^ cells were washed in PBS and resuspended in a 100 µL fluorescence-activated cell sorting (FACS) buffer with 5 µL antibody and incubated at 4 °C for 1 h. Moreover, single cell suspension of the tumor cells from CRC patients was obtained as described previously ^4^, followed by incubation with mouse anti-human HER2/Neu antibody and flow cytometry analysis. Appropriate isotype controls were also utilized. Stained cells were washed twice with the FACS buffer. The acquired data were analyzed using FlowJo.V10 software (Tree Star, Ashland, OR, USA).

**CAR T-cell cytotoxicity and cytokine secretion assays**

The density of the target cells (DLD-1) was adjusted to 5 × 10^5^/mL and maintained in RPMI 1640 medium containing 10% (v/v) FBS, 100 U/mL penicillin, and 100 μg/mL streptomycin in 96-well plates (100 µL/well; Corning Incorporated). After overnight incubation, the plates were washed, the CAR T cells were resuspended in FBS-free RPMI 1640 medium and added at 1:1, 5:1, 10:1 T cell:target cell ratio to each well, the final volume was adjusted to 100 µL/well, and cultured at 37 °C in 5% CO_2_. After 6 h of co-culture, 50 µL supernatant was collected by centrifugation for lactate dehydrogenase (LDH) detection using the LDH release kit (Cat: G1780, Promega, Madison, WI, USA), according to the manufacturer’s instructions. The level of LDH was calculated as follows: Lysis% = ([OD of each well - OD of mini lysis]) / (OD of maxi lysis) × 100%. Moreover, at 24 h after co-incubation, the remaining 50 µL supernatant was also collected for the detection of IL-2 by human IL-2 ELISA kit (MultiSciences, Hangzhou, China).

**Apoptosis assay**

1 × 10^5^ cells were washed twice with PBS and resuspended in 100 µL 1X binding buffer according to the instructions of apoptosis detection kit (Cat: 556547, BD Biosciences). As described elsewhere ^5^, a 5 µL volume of fluorescein isothiocyanate (FITC)-conjugated Annexin-V and Propidium Iodide (PI) was added to the cells, and the reaction incubated at RT in the dark for 15 min. A volume of 200 µL of 1X binding buffer was added to the cells, followed by flow cytometry. The percentage of apoptotic cells was calculated based on the Annexin-V+/PI+ double-stained population.

**Karyotype analysis**

As described previously ^6^, T cells were collected on the 14th day after lentivirus infection. Briefly, when the number of T cells reached 1 × 10^6^, colcemid (XP Biomed, Shanghai, China) with the final concentration of 0.1 μg/mL was added to the cell culture medium, and the cells were put back into the incubator for another 4 h so that the cell division stopped in the middle stage. A centrifuge was used to collect the cells, and then add the 0.075 mol/L KCl (BBI Solutions, Madison, WI, USA) of 5 mL at 37 °C for 30 min. Next, the cells were fixed in a freshly prepared fixed solution (methanol / acetic acid = 3: 1, v/v), 2-3 drops of cell suspension were dropped on the slide, and then stained with pH7.4 phosphate buffer (Gibco) diluted Giemsa solution (9:1, v/v) (Mybioscience, Paris, France) for 15-20 min, rinsed with clean water and dried at RT. Karyotype analysis of chromosome images was performed on VideoTesT-Karyo system (internet Microscope Technology, Brooklyn, NY, USA).

**Statistical analysis**

Data are expressed as the mean ± standard error of the mean. The Wilcoxon test was used to assess the differences in HER2 staining scores in the TMA. The Log-rank test was used to analyze the survival curves. The values of flank tumor and metastatic liver and lung burdens in mice were determined by two-tailed t-test. Statistical analysis was performed using SPSS 12.0, and the differences were considered significant at *P* < 0.05. Histograms were constructed using GraphPad Prism Software (Version 6.04).

References

1. Meng Q*, et al.* MPO Promoter Polymorphism rs2333227 Enhances Malignant Phenotypes of Colorectal Cancer by Altering the Binding Affinity of AP-2alpha. *Cancer Res* 2018, **78**(10)**:** 2760-2769.

2. Zhang C*, et al.* Role of astrocyte activation in fine particulate matter-enhancement of existing ischemic stroke in Sprague-Dawley male rats. *Journal of toxicology and environmental health Part A* 2016, **79**(9-10)**:** 393-401.

3. van der Pool AE*, et al.* Trends in incidence, treatment and survival of patients with stage IV colorectal cancer: a population-based series. *Colorectal disease : the official journal of the Association of Coloproctology of Great Britain and Ireland* 2012, **14**(1)**:** 56-61.

4. Qu L*, et al.* Exosome-Transmitted lncARSR Promotes Sunitinib Resistance in Renal Cancer by Acting as a Competing Endogenous RNA. *Cancer cell* 2016, **29**(5)**:** 653-668.

5. Daneshmanesh AH*, et al.* Monoclonal antibodies against ROR1 induce apoptosis of chronic lymphocytic leukemia (CLL) cells. *Leukemia* 2012, **26**(6)**:** 1348-1355.

6. Chou CH*, et al.* Chromosome instability modulated by BMI1-AURKA signaling drives progression in head and neck cancer. *Cancer Res* 2013, **73**(2)**:** 953-966.

Table S1. Relationship between expression levels of HER2 and selected clinic pathologic features of the CRC patients

| Variables | Xuzhou cohort (n=360) | | |  | Nanjing cohort (n=680) | | |  |  | combined cohort (n=1040) | | |
| --- | --- | --- | --- | --- | --- | --- | --- | --- | --- | --- | --- | --- |
|  | Low (%) | High (%) | *P* |  | Low (%) | High (%) | *P* |  |  | Low (%) | High (%) | *P* |
| All patients | 235(65.3) | 125(34.7) |  |  | 441(64.9) | 239(35.2) |  |  |  | 676(65.0) | 364(35.0) |  |
| Age |  |  | 0.9804 |  |  |  | 0.4005 |  |  |  |  | 0.5030 |
| ≤55 | 120(51.1) | 64(51.2) |  |  | 216(49.0) | 109(45.6) |  |  |  | 336(49.7) | 173(47.5) |  |
| ＞55 | 115(48.9) | 61(48.8) |  |  | 225(51.0) | 130(54.4) |  |  |  | 340(50.3) | 191(52.5) |  |
| Gender |  |  | 0.2253 |  |  |  | 0.1196 |  |  |  |  | 0.0483 |
| Males | 133(56.6) | 79(63.2) |  |  | 261(59.2) | 156(65.3) |  |  |  | 394(58.3) | 235(64.6) |  |
| Females | 102(43.4) | 46(36.8) |  |  | 180(40.8) | 83(34.7) |  |  |  | 282(41.7) | 129(35.4) |  |
| Location |  |  | 0.6284 |  |  |  | 0.6443 |  |  |  |  | 0.9270 |
| Colon | 104(44.3) | 52(41.6) |  |  | 193(43.8) | 109(45.6) |  |  |  | 297(43.9) | 161(44.2) |  |
| Rectal | 131(55.7) | 73(58.4) |  |  | 248(56.2) | 130(54.4) |  |  |  | 379(56.1) | 203(55.8) |  |
| Grade |  |  | 0.0028 |  |  |  | 0.5354 |  |  |  |  | 0.0241 |
| low | 65(27.7) | 54(43.2) |  |  | 141(32.0) | 82(34.3) |  |  |  | 206(30.5) | 136(37.4) |  |
| Intermediate/High | 170(72.3) | 71(56.8) |  |  | 300(68.0) | 157(65.7) |  |  |  | 470(69.5) | 228(62.6) |  |
| Depth of invasion |  |  | 0.7677 |  |  |  | 0.0036 |  |  |  |  | 0.0064 |
| T1 | 10(4.3) | 4(3.2) |  |  | 18(4.1) | 3(1.3) |  |  |  | 28(4.1) | 7(1.9) |  |
| T2 | 50(21.3) | 22(17.6) |  |  | 114(25.9) | 49(20.5) |  |  |  | 164(24.3) | 71(19.5) |  |
| T3 | 42(17.9) | 22(17.6) |  |  | 94(21.3) | 38(15.9) |  |  |  | 136(20.1) | 60(16.5) |  |
| T4 | 133(56.6) | 77(61.6) |  |  | 215(48.8) | 149(62.3) |  |  |  | 348(51.5) | 226(62.1) |  |
| Lymph node metastasis |  |  | 0.0001 |  |  |  | <.0001 |  |  |  |  | <.0001 |
| N0 | 142(60.4) | 49(39.2) |  |  | 312(70.8) | 102(42.7) |  |  |  | 454(67.2) | 151(41.5) |  |
| N1 | 93(39.6) | 76(60.8) |  |  | 129(29.3) | 137(57.3) |  |  |  | 222(32.8) | 213(58.5) |  |
| Distant metastasis |  |  | 0.0051 |  |  |  | <.0001 |  |  |  |  | <.0001 |
| M0 | 214(91.1) | 101(80.8) |  |  | 416(94.3) | 193(80.8) |  |  |  | 630(93.2) | 294(80.8) |  |
| M1 | 21(8.9) | 24(19.2) |  |  | 25(5.7) | 46(19.3) |  |  |  | 46(6.8) | 70(19.2) |  |
| TNM |  |  | 0.0001 |  |  |  | <.0001 |  |  |  |  | <.0001 |
| I | 39(16.6) | 14(11.2) |  |  | 111(25.2) | 27(11.3) |  |  |  | 150(22.2) | 41(11.3) |  |
| II | 101(43.0) | 30(24.0) |  |  | 194(44.0) | 65(27.2) |  |  |  | 295(43.6) | 95(26.1) |  |
| III | 74(31.5) | 57(45.6) |  |  | 111(25.2) | 101(42.3) |  |  |  | 185(27.4) | 158(43.4) |  |
| IV | 21(8.9) | 24(19.2) |  |  | 25(5.7) | 46(19.3) |  |  |  | 46(6.8) | 70(19.2) |  |

Table S2. Demographic information of CRC for HER2 protein expression by flow cytometry

| Variables | Jiangsu Tumor Hospital  (n=75) | Jiangsu Province Hospital  (n=31) |
| --- | --- | --- |
| Age |  |  |
| <62 | 34(45.3) | 10 (32.3) |
| ≥62 | 41(54.7) | 21 (67.7) |
| Gender |  |  |
| Males | 42 (56.0) | 23 (74.2) |
| Females | 33 (44.0) | 8 (25.8) |
| Location |  |  |
| Colon | 34 (45.3) | 12 (38.7) |
| Rectal | 41 (54.7) | 19 (61.3) |
| Depth of invasion |  |  |
| T1 | 9 (12.0) | 3 (9.7) |
| T2 | 10 (13.3) | 6 (19.4) |
| T3 | 35 (46.7) | 16 (51.6) |
| T4 | 21 (28.0) | 6 (19.4) |
| Lymph node metastasis |  |  |
| N0 | 37 (49.3) | 16 (51.6) |
| N1 | 38 (50.7) | 15 (48.4) |
| Distant metastasis |  |  |
| M0 | 68 (90.7) | 25 (80.6) |
| M1 | 7 (9.3) | 6 (19.4) |
| TNM |  |  |
| I | 14 (18.7) | 9 (29.0) |
| II | 20 (26.7) | 7 (22.6) |
| III | 34 (45.3) | 9 (29.0) |
| IV | 7 (9.3) | 6 (19.4) |

Table S3. Multivariate Cox regression analysis of HER2 expression and selected clinic pathologic variables with CRC survival

| Variables | Xuzhou cohort (n=360) | |  | Nanjing cohort (n=680) | |  | combined cohort (n=1040) | |
| --- | --- | --- | --- | --- | --- | --- | --- | --- |
|  | Adjusted HR(95%CI) ^a^ | *P* ^a^ |  | Adjusted HR(95%CI) ^a^ | *P* ^a^ |  | Adjusted HR(95%CI) ^a^ | *P* ^a^ |
| Age (＞55 VS. ≤55) | 1.27(0.98-1.64) | 0.0723 |  | 1.56(1.27-1.92) | <.0001 |  | 1.45(1.23-1.70) | <.0001 |
| Gender (Females VS. Males) | 0.91(0.70-1.19) | 0.4871 |  | 0.95(0.77-1.18) | 0.6323 |  | 0.93(0.79-1.10) | 0.3888 |
| Location (Rectum VS. Colon) | 0.86(0.67-1.11) | 0.2547 |  | 0.72(0.59-0.88) | 0.0015 |  | 0.77(0.65-0.90) | 0.0012 |
| Grade (Intermediate/High VS. Low) | 0.89(0.68-1.17) | 0.4046 |  | 0.95(0.77-1.18) | 0.6394 |  | 0.95(0.80-1.12) | 0.5415 |
| TNM (III/IV VS. I/II) | 4.42(3.32-5.88) | <.0001 |  | 5.59(4.45-7.01) | <.0001 |  | 5.16(4.33-6.16) | <.0001 |
| HER2 (High VS. Low) | 1.83(1.40-2.40) | <.0001 |  | 2.68(2.17-3.30) | <.0001 |  | 2.34(1.98-2.75) | <.0001 |
| ^a^ Adjusted for age, gender, location(Colon or Rectum), grade (Low VS. Intermediate/High), clinical stage (I/II VS. III/IV) and HER2 expression. | | | | | | | | |

Table S4. Demographic characteristics of CRC patients recruited for PDX model

| Samples | Gender | Age(years) | TNM stage | 7th Edition AJCC | Location | Diagnosis |
| --- | --- | --- | --- | --- | --- | --- |
| CRC-1 | Male | 62 | T4N0M1 | Ⅳ | Colon | CRC with multiple hepatic metastasis |
| CRC-2 | Male | 53 | T4N1M1 | Ⅳ | Colon | CRC with pulmonary metastasis |
| CRC-3 | Male | 49 | T4N1M1 | Ⅳ | Colon | CRC with hepatic metastasis |
| CRC-4 | Female | 73 | T4N0M1 | Ⅳ | Colon | CRC with hepatic metastasis |
| CRC-5 | Male | 66 | T3N1M1 | Ⅳ | Colon | CRC with pulmonary metastasis |
| CRC-8 | Male | 60 | T4N0M1 | Ⅳ | Rectal | CRC with pulmonary metastasis |
| CRC-6 | Female | 48 | T4N2M0 | Ⅲ | Rectal | CRC |
| CRC-7 | Male | 52 | T4N2M0 | Ⅲ | Rectal | CRC |
| CRC-9 | Female | 54 | T3N2M0 | Ⅲ | Rectal | CRC |
| CRC-10 | Female | 71 | T3N2M0 | Ⅲ | Colon | CRC |
| CRC-11 | Female | 83 | T3N2M0 | Ⅲ | Rectal | CRC |
| CRC-12 | Female | 66 | T3N2M0 | Ⅲ | Colon | CRC |

**Supplementary Figure Legends**

**Supplementary Figure S1–S5. A total of 106 pairs of fresh CRC tumors and matched adjacent tissues were measured by flow cytometry.**

**Supplementary Figure S6. Construction of HER2-specific CAR.**
